# Comparing Trabeculectomy Outcomes between First and Second Operated Eyes: A Multicenter Study

**DOI:** 10.1371/journal.pone.0162569

**Published:** 2016-09-13

**Authors:** Kentaro Iwasaki, Yoshihiro Takamura, Takashi Nishida, Akira Sawada, Keiichiro Iwao, Ayano Shinmura, Shiho Kunimatsu-Sanuki, Tetsuya Yamamoto, Hidenobu Tanihara, Kazuhisa Sugiyama, Toru Nakazawa, Masaru Inatani

**Affiliations:** 1 Department of Ophthalmology, Faculty of Medical Sciences, University of Fukui, Fukui, Japan; 2 Department of Ophthalmology, Gifu University Graduate School of Medicine, Gifu, Japan; 3 Department of Ophthalmology, Faculty of Life Sciences, Kumamoto University, Kumamoto, Japan; 4 Department of Ophthalmology and Visual Science, Kanazawa University Graduate School of Medical Science, Kanazawa, Japan; 5 Department of Ophthalmology, Tohoku University Graduate School of Medicine, Sendai, Japan; Massachusetts Eye & Ear Infirmary, Harvard Medical School, UNITED STATES

## Abstract

**Objective:**

To compare surgical outcomes between the first and second operated eyes in patients who underwent trabeculectomy in both eyes.

**Methods:**

This retrospective clinical cohort study at five clinical centers in Japan included 84 patients with open-angle glaucoma who underwent primary trabeculectomy in both eyes. The primary outcome was surgical success or failure, with failure being defined according to three criteria: <20% reduction of the preoperative intraocular pressure (IOP), or Criterion A, IOP >21 mmHg; Criterion B, IOP >18 mmHg; or Criterion C, IOP >15 mmHg. Cases of reoperation, a loss of light perception vision, or hypotony were also considered as “failures”.

**Results:**

There were no significant differences in success rate for any of the three criteria between the first and second operated eyes. For patients whose first trabeculectomy was successful, when the second trabeculectomy was performed ≥2 months after the first, the survival curves for all three criteria for the second trabeculectomy were significantly worse than those for patients waiting a shorter interval between trabeculectomies (Criterion A, 52.0% vs 83.6%, P = 0.0031; Criterion B, 51.5% vs 80.4%, P = 0.026; Criterion C, 51.1% vs 80.4%, P = 0.048). In multivariable analyses, a longer interval between trabeculectomies was a significant prognostic factor for surgical failure (Criterion A, P = 0.0055; Criterion B, P = 0.0023; Criterion C, P = 0.027). However, no dependency on the interval between trabeculectomies was found among patients whose first trabeculectomy failed.

**Conclusions:**

If the first trabeculectomy is successful, a long interval before the second trabeculectomy increases the risk of surgical failure in the second eye. This result has clinical implications for developing surgical strategies for patients with bilateral glaucoma.

## Introduction

Trabeculectomy is the most commonly used surgical procedure for lowering intraocular pressure (IOP) in eyes with glaucoma. Reduction of the IOP is achieved by creating a surgical bypass for aqueous humor from the anterior chamber to the conjunctiva via the scleral flap, creating a filtering bleb in the conjunctiva [1],[2],[3]. Risks for surgical failure of trabeculectomy include perioperative inflammation, which appears to be associated with bleb failure in trabeculectomized eyes because of uveitic glaucoma [4],[5], trabeculectomy with combined lens extraction [6],[7],[8], and elevation of inflammatory cytokines in the aqueous humor [9]. Ocular inflammation recruits leukocytes in the conjunctiva, which promote fibrosis in the filtering bleb [10]. In addition, because aqueous humor delivers intraocular antigens to the ocular surface through the bypass in the scleral flap after trabeculectomy, patients with filtering blebs seem to systemically acquire immunity to intraocular antigens [11].

Eyes in which filtering surgery, including trabeculectomy and tube-shunt surgery, have been performed are at risk for corneal transplantation graft failure [12],[13],[14]. It has been hypothesized that this may be caused by a change in immune status after the creation of a communication between the anterior chamber and the subconjunctival space [12]. Thus, if both of a patient’s eyes are treated with trabeculectomy, the immune communication between the intraocular antigens and the subconjunctival space in the first operated eye might affect bleb formation in the second operated eye. There have been two large-scale retrospective studies about trabeculectomy for both eyes [15],[16]. These found no significant difference in surgical success between the first and second operated eyes. However, bleb needling and revision occurred more frequently in the second operated eyes, indicating the possibility of an immune reaction affected by the first operated eyes [15].

To determine whether the surgical outcome for trabeculectomy of second operated eyes is worse than that for first operated eyes, we analyzed retrospective data for patients with open-angle glaucoma (OAG) who were treated with trabeculectomy in both eyes at five clinical centers in Japan.

## Materials and Methods

### Patient Selection

This retrospective clinical cohort study was approved by the institutional review board of Fukui University Hospital, Fukui, Japan. The protocol adhered to the tenets of the Declaration of Helsinki. Written informed consent for the surgery was obtained from all patients after a detailed explanation of the procedures involved.

Patients treated with trabeculectomy for their second operated eyes between January 1, 2007 and December 31, 2011 at Fukui University Hospital, Gifu University Hospital, Kumamoto University Hospital, Kanazawa University Hospital and Tohoku University Hospital in Japan were recruited. The inclusion criteria were being aged 20 years or older, having primary OAG or exfoliation glaucoma, and having both eyes operated by the same surgeon. The exclusion criteria were as follows: previous vitreoretinal surgery, including vitrectomy and buckling surgery; previous glaucoma surgery; having one phakic eye and one pseudophakic eye before trabeculectomy; or having one fornix-based and one limbus-based trabeculectomized eye. Mitomycin C was used intraoperatively in all the trabeculectomies.

### Data Collection

Patient data were collected for the subjects, including sex, age, type of glaucoma, lens status, preoperative IOP, postoperative IOP, best corrected visual acuity (BCVA), the number of glaucoma medications taken, and any postoperative complications. A logarithm of the reciprocal of the decimal BCVA was used to approximate the logarithm of the minimal angle of resolution (logMAR).

### Primary Outcome Measure

The primary outcome measure was surgical success or failure defined according to three IOP criteria. The criteria for failure were the following IOP levels, with or without glaucoma medication, at ≥3 months after surgery and confirmed 1 month later: <20% reduction of the preoperative IOP, or Criterion A, IOP >21 mmHg; Criterion B, IOP >18 mmHg; or Criterion C, IOP >15 mmHg. In addition, surgical failure was declared for all criteria in cases that required reoperation for glaucoma or that developed a loss of light perception vision or encountered hypotony of ≤5 mmHg. In cases that did not meet these failure criteria, the surgery was considered to be successful. The probability of success was compared between the first and second operated eyes.

### Secondary Outcome Measures

Secondary outcome measures included IOP, the number of medications taken, and postoperative complications.

### Statistical Analysis

Univariable comparisons between groups were performed using the chi-square test, Fisher’s exact test, and the Mann–Whitney U nonparametric test. The probability of success was analyzed using Kaplan–Meier survival curves and compared using the log-rank test. P values of <0.05 were considered to be statistically significant. Multivariable analysis was performed to determine the prognostic factors for failure of trabeculectomy using Cox proportional hazards models.

## Results

### Patients Characteristics

In total, 84 patients were enrolled in the study. The mean follow-up periods were 50.0 months for the first operated eyes and 43.2 months for the second operated eyes (P = 0.066). Table 1 summarizes the baseline characteristics of the patients, and Table 2 presents the preoperative characteristics for the first and second operated eyes. Best-corrected visual acuity (LogMAR) of the first operated eyes was significantly worse than that of the second operated eyes (P = 0.017). No other statistically significant differences in preoperative status were found between the eyes.

**Table 1:** Preoperative patient characteristics.

Characteristics	n (%), total n = 84
Sex	
Men	54 (64%)
Women	30 (36%)
Lens status	
Phakic	70 (83%)
Pseudophakic	14 (17%)
Type of glaucoma	
Primary open-angle glaucoma	68 (81%)
Exfoliation glaucoma	16 (19%)
Conjunctival approach	
Limbus-based	36 (43%)
Fornix-based	48 (57%)

**Table 2:** Preoperative characteristics for the first and second operated eyes.

	1^st^ operated eye	2^nd^ operated eye	P value
Age (years)	66.9 ± 12.8	67.8 ± 12.6	0.56
LogMAR BCVA	0.44 ± 0.70	0.18 ± 0.46	0.017*
IOP (mmHg)	23.9 ± 9.5	21.1 ± 6.5	0.12
Number of glaucoma medications	3.1 ± 0.6	3.1 ± 0.8	0.92
Follow-up period (months)	50.0 ± 24.2	43.2 ± 23.0	0.066

Data are presented as mean ± standard deviation.

*Statistically significant difference, P < 0.05.

BCVA = best-corrected visual acuity; IOP = intraocular pressure; logMAR = logarithm of minimum angle of resolution

### Primary Outcome Measure

**Fig 1:**
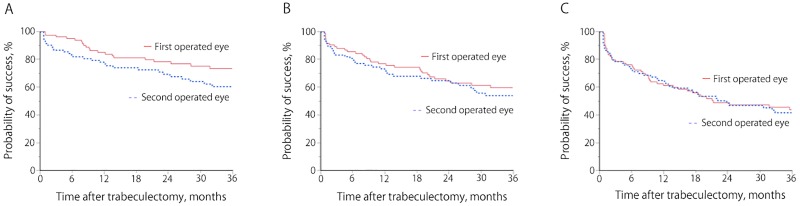
Kaplan–Meier survival curves for the three failure criteria, comparing the first and second operated eyes. Criterion A: intraocular pressure (IOP) >21 mmHg, <20% reduction of preoperative IOP, reoperation for glaucoma, a loss of light perception vision, or hypotony of ≤5 mmHg. Criterion B: IOP >18 mmHg, <20% reduction of preoperative IOP, reoperation for glaucoma, a loss of light perception vision, or hypotony of ≤5 mmHg. Criterion C: IOP >15 mmHg, <20% reduction of preoperative IOP, reoperation for glaucoma, a loss of light perception vision, or hypotony of ≤5 mmHg. The cumulative success rates for first and second operated eyes were 73.6% and 60.6% for Criterion A (*P* = 0.090), 59.8% and 54.0% for Criterion B (*P* = 0.42), and 43.8% and 41.7% for Criterion C (*P* = 0.57).

Kaplan–Meier survival curves comparing surgical outcomes in the first and second operated eyes according to failure criteria A, B, and C are shown in Fig 1. No significant difference between the two eyes was found for any criterion. The probability of success at 3 years for the first versus the second operated eyes was 73.6% vs 60.6% for Criterion A (*P* = 0.090), 59.8% vs 54.0% for Criterion B (*P* = 0.42), and 43.8% vs 41.7% for Criterion C (*P* = 0.57).

### Secondary Outcome Measures

IOPs and the number of glaucoma medications being taken at various follow-up time points were compared between the first and second operated eyes (Table 3). No significant differences in these outcomes were found at any time points after trabeculectomy. Table 4 compares postoperative complications between the eyes; there was no significant difference.

**Table 3:** Intraocular pressure and medical therapy at preoperative and follow-up visits.

	1^st^ operated eye	2^nd^ operated eye	P value
Preoperative			
IOP (mm Hg)	23.9 ± 9.5	21.1 ± 6.5	0.12
Number of mediation	3.1 ± 0.6	3.1 ± 0.8	0.92
Number of patients	84	84	
6M			
IOP (mm Hg)	11.0 ± 4.7	11.2 ± 4.3	0.78
Number of mediation	0.1 ± 0.5	0.3 ± 0.7	0.22
Number of patients	83	84	
12M			
IOP (mm Hg)	11.9 ± 4.3	12.3 ± 4.6	0.87
Number of mediation	0.3 ± 0.8	0.4 ± 0.9	0.35
Number of patients	79	77	
18M			
IOP (mm Hg)	12.7 ± 4.9	13.4 ± 5.4	0.63
Number of mediation	0.5 ± 0.9	0.6 ± 1.0	0.39
Number of patients	75	70	
24M			
IOP (mm Hg)	12.9 ± 4.6	12.9 ± 4.7	0.80
Number of mediation	0.7 ± 1.1	0.8 ± 1.1	0.63
Number of patients	71	64	
30M			
IOP (mm Hg)	13.0 ± 4.2	12.8 ± 3.9	0.85
Number of mediation	0.8 ± 1.2	0.9 ± 1.2	0.98
Number of patients	62	55	
36M			
IOP (mm Hg)	12.4 ± 4.2	12.3 ± 4.1	0.78
Number of mediation	0.9 ± 1.3	1.0 ± 1.4	0.93
Number of patients	58	50	

Data shown in mean ± standard deviation; IOP = intraocular pressure

**Table 4:** Postoperative complications of the 1^st^-operated eyes and 2^nd^-operated eyes.

	1^st^ operated eye (n = 84)	2^nd^ operated eye (n = 84)	P value
Bleb leak	5 (6.0%)	4 (4.8%)	1.00
Bleb infection	2 (2.4%)	1 (1.2%)	1.00
Hyphema	3 (3.6%)	6 (7.1%)	0.50
Choroidal detachment	12 (14.3%)	8 (9.5%)	0.48
Shallow anterior chamber	5 (6.0%)	4 (4.8%)	1.00
Hypotony maculopathy	0 (0.0%)	1 (1.2%)	1.00
Malignant glaucoma	1 (1.2%)	0 (0.0%)	1.00

### Subgroup Analyses of the Primary Outcome

For patients whose first operation was successful, characteristics were compared between those whose second trabeculectomy was successful and those for whom it failed (Tables 5–7). The interval between trabeculectomies was significantly longer for the failed second operated eyes than for those that were successful, with the mean intervals being 574 vs 180 days for Criterion A (P = 0.0030), 518 vs 156 days for Criterion B (P = 0.0036), and 508 vs 148 days for Criterion C (P = 0.015). No significant difference in age, preoperative IOP, number of preoperative glaucoma medications, type of glaucoma, or lens status was found for any of the three criteria.

**Table 5:** The comparison of the successful 2^nd^ operated eyes versus failed 2^nd^ operated eyes among successful 1^st^ operated eyes, Criterion A: n = 57.

	Successful 2^nd^ operated eye (n = 40)	Failed 2^nd^ operated eye (n = 17)	P value
Interval between TLE (days), mean ± SD	180 ± 299	574 ± 602	0.0030
Age (years), mean ± SD	70.0 ± 13.0	65.9 ± 11.2	0.17
Preoperative IOP (mmHg), mean ± SD	22.7 ± 6.8	19.9 ± 6.1	0.14
Preoperative glaucoma medications, mean ± SD	2.9 ± 0.9	2.9 ± 0.6	0.77
Type of glaucoma, n (%)			1.00
Primary open-angle glaucoma	32 (80.0%)	14 (82.4%)	
Exfoliation glaucoma	8 (20.0%)	3 (17.6%)	
Lens status, n (%)			1.00
Phakic	34 (85.0%)	14 (82.3%)	
Pseudophakic	6 (15.0%)	3 (17.7%)	

IOP = intraocular pressure; SD = standard deviation; TLE = trabeculectomy

**Table 6:** The comparison of the successful 2^nd^ operated eyes versus failed 2^nd^ operated eyes among successful 1^st^ operated eyes, Criterion B: n = 42.

	Successful 2^nd^ operated eye (n = 28)	Failed 2^nd^ operated eye (n = 14)	P value
Interval between TLE (days), mean ± SD	156 ± 223	518 ± 441	0.0036
Age (years), mean ± SD	70.3 ± 13.1	65.3 ± 12.0	0.11
Preoperative IOP (mmHg), mean ± SD	22.0 ± 7.6	19.3 ± 6.1	0.28
Preoperative glaucoma medications, mean ± SD	3.1 ± 0.9	3.0 ± 0.6	0.57
Type of glaucoma, n (%)			1.00
Primary open-angle glaucoma	22 (78.6%)	11 (78.6%)	
Exfoliation glaucoma	6 (21.4%)	3 (21.4%)	
Lens status, n (%)			1.00
Phakic	24 (85.7%)	12 (85.7%)	
Pseudophakic	4 (14.3%)	2 (14.3%)	

IOP = intraocular pressure; SD = standard deviation; TLE = trabeculectomy

**Table 7:** The comparison of the successful 2^nd^ operated eyes versus failed 2^nd^ operated eyes among successful 1^st^ operated eyes, Criterion C: n = 30.

	Successful 2^nd^ operated eye (n = 21)	Failed 2^nd^ operated eye (n = 9)	P value
Interval between TLE (days), mean ± SD	148 ± 236	508 ± 478	0.015
Age (years), mean ± SD	67.5 ± 13.0	62.9 ± 8.0	0.093
Preoperative IOP (mmHg), mean ± SD	19.6 ± 6.1	16.3 ± 3.8	0.17
Preoperative glaucoma medications, mean ± SD	3.1 ± 0.9	2.8 ± 0.4	0.12
Type of glaucoma, n (%)			0.53
Primary open-angle glaucoma	18 (85.7%)	9 (100%)	
Exfoliation glaucoma	3 (14.3%)	0 (0.0%)	
Lens status, n (%)			1.00
Phakic	20 (95.2%)	9 (100%)	
Pseudophakic	1 (4.8%)	0 (0.0%)	

IOP = intraocular pressure; SD = standard deviation; TLE = trabeculectomy

A similar analysis was performed for patients whose trabeculectomy in the first eye failed (Tables 8–10). No significant difference in the interval between trabeculectomies, age, preoperative IOP, number of preoperative glaucoma medications, type of glaucoma, or lens status was found for any of the three criteria.

**Table 8:** The comparison of the successful 2^nd^ operated eyes versus failed 2^nd^ operated eyes among failed 1^st^ operated eyes, Criterion A: n = 27.

	Successful 2^nd^ operated eye (n = 11)	Failed 2^nd^ operated eye (n = 16)	P value
Interval between TLE (days), mean ± SD	496 ± 753	280 ± 817	0.33
Age (years), mean ± SD	65.9 ± 18.4	65.5 ± 7.3	0.36
Preoperative IOP (mmHg), mean ± SD	19.6 ± 4.5	19.6 ± 6.8	0.40
Preoperative glaucoma medications, mean ± SD	3.3 ± 0.5	3.4 ± 0.7	0.73
Type of glaucoma, n (%)			1.00
Primary open-angle glaucoma	9 (81.8%)	13 (81.2%)	
Exfoliation glaucoma	2 (18.2%)	3 (18.8%)	
Lens status, n (%)			0.37
Phakic	8 (72.7%)	14 (87.5%)	
Pseudophakic	3 (27.3%)	2 (12.5%)	

IOP = intraocular pressure; SD = standard deviation; TLE = trabeculectomy

**Table 9:** The comparison of the successful 2^nd^ operated eyes versus failed 2^nd^ operated eyes among failed 1^st^ operated eyes, Criterion B: n = 42.

	Successful 2^nd^ operated eye (n = 20)	Failed 2^nd^ operated eye (n = 22)	P value
Interval between TLE (days), mean ± SD	464 ± 726	273 ± 742	0.28
Age (years), mean ± SD	68.8 ± 15.1	65.3 ± 9.5	0.12
Preoperative IOP (mmHg), mean ± SD	22.0 ± 5.4	20.5 ± 6.1	0.21
Preoperative glaucoma medications, mean ± SD	3.0 ± 0.6	3.1 ± 1.0	0.33
Type of glaucoma, n (%)			1.00
Primary open-angle glaucoma	17 (85.0%)	18 (81.8%)	
Exfoliation glaucoma	3 (15.0%)	4 (18.2%)	
Lens status, n (%)			0.45
Phakic	15 (75.0%)	19 (86.4%)	
Pseudophakic	5 (25.0%)	3 (13.6%)	

IOP = intraocular pressure; SD = standard deviation; TLE = trabeculectomy

**Table 10:** The comparison of the successful 2^nd^ operated eyes versus failed 2^nd^ operated eyes among failed 1^st^ operated eyes, Criterion C: n = 54.

	Successful 2^nd^ operated eye (n = 24)	Failed 2^nd^ operated eye (n = 30)	P value
Interval between TLE (days), mean ± SD	487 ± 674	251 ± 645	0.076
Age (years), mean ± SD	69.5 ± 15.3	68.1 ± 11.2	0.32
Preoperative IOP (mmHg), mean ± SD	23.1 ± 6.4	22.1 ± 6.6	0.31
Preoperative glaucoma medications, mean ± SD	3.2 ± 0.6	3.0 ± 0.9	0.61
Type of glaucoma, n (%)			1.00
Primary open-angle glaucoma	18 (75.0%)	23 (76.7%)	
Exfoliation glaucoma	6 (25.0%)	7 (23.3%)	
Lens status, n (%)			0.53
Phakic	17 (70.8%)	24 (80.0%)	
Pseudophakic	7 (29.2%)	6 (20.0%)	

IOP = intraocular pressure; SD = standard deviation; TLE = trabeculectomy

**Fig 2:**
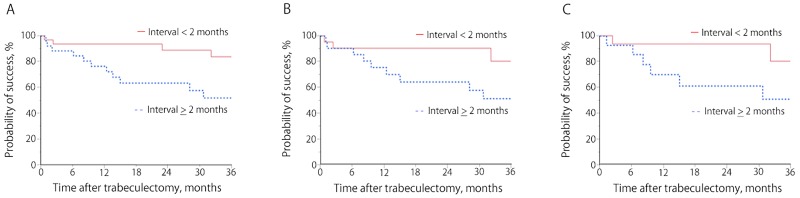
Kaplan–Meier survival curves for each of the three failure criteria in the second operated eye, comparing patients for whom the interval between trabeculectomies was ≥2 months with those for whom the interval was <2 months. Criterion A: intraocular pressure (IOP) >21 mmHg, <20% reduction of preoperative IOP, reoperation for glaucoma, a loss of light perception vision, or hypotony of ≤5 mmHg. Criterion B: IOP >18 mmHg, <20% reduction of preoperative IOP, reoperation for glaucoma, a loss of light perception vision, or hypotony of ≤5 mmHg. Criterion C: IOP >15 mmHg, <20% reduction of preoperative IOP, reoperation for glaucoma, a loss of light perception vision, or hypotony of ≤5 mmHg. The cumulative success rates for intervals between trabeculectomies of ≥2 months and <2 months were 52.0% and 83.6% for Criterion A (*P* = 0.0031), 51.5% and 80.4% for Criterion B (*P* = 0.026), and 51.1% and 80.4% for Criterion C (*P* = 0.048).

Fig 2 shows the Kaplan–Meier survival curves for the three criteria applied to the second operated eye, comparing patients for whom the interval between trabeculectomies was ≥2 months with those for whom the interval was <2 months. The outcomes were significantly worse for the patients where the interval was ≥2 months, with the success rates at 3 years being 52.0% vs 83.6% for Criterion A (P = 0.0031), 51.5% vs 80.4% for Criterion B (P = 0.026), and 51.1% vs 80.4% for Criterion C (P = 0.048).

### Multivariable analysis to determine prognostic factors for the surgical failure of trabeculectomy

Baseline characteristics, including age, type of glaucoma, preoperative IOP, lens status, the number of preoperative glaucoma medication taken, and the interval between trabeculectomies were evaluated as possible predictors of surgical failure.

In analyses using multivariable Cox proportional hazards regression models for patients whose first trabeculectomies were successful (Table 11), a longer interval between trabeculectomies was a significant prognostic factor for surgical failure in the second operated eye for all three criteria: Criterion A, relative risk (RR) = 1.07 (P = 0.0055); Criterion B, RR = 1.12 (P = 0.0023); and Criterion C, RR = 1.10 (P = 0.027). In contrast, in the multivariable analyses of patients whose first trabeculectomies failed (Table 12), no significant difference was found for any factor.

**Table 11:** Multivariable analysis to determine prognostic factors for surgical failure of 2^nd^ operated trabeculectomy using Cox proportional hazards regression models among the successful 1^st^ operated eyes.

			Criterion			
	A		B		C	
	RR (95% Cl)	P value	RR (95% Cl)	P value	RR (95% Cl)	P value
Interval between TLE per month	1.07 (1.02–1.13)	<0.01	1.12 (1.04–1.24)	<0.01	1.10 (1.01–1.23)	0.027
Preoperative IOP per mmHg	0.94 (0.83–1.05)	0.28	0.90 (0.76–1.04)	0.15	0.94 (0.74–1.15)	0.54
Age per year	0.97 (0.91–1.04)	0.38	0.99 (0.92–1.07)	0.71	0.99 (0.92–1.10)	0.88
Type of glaucoma (XFG/POAG)	0.99 (0.12–6.42)	0.99	1.62 (0.18–14.4)	0.65	0.00 (0.00–3.37)	0.18
Lens status (pseudophakia/phakia)	2.93 (0.31–28.5)	0.34	3.83 (0.20–83.7)	0.36	0.00 (0.00–216)	0.69
Preoperative glaucoma medication per each	1.37 (0.61–3.82)	0.47	1.16 (0.42–4.20)	0.79	0.74 (0.22–2.44)	0.58

IOP = intraocular pressure; POAG = primary open-angle glaucoma; RR = relative risk; TLE = trabeculectomy; XFG = exfoliation glaucoma

**Table 12:** Multivariable analysis to determine prognostic factors for surgical failure of 2^nd^ operated trabeculectomy using Cox proportional hazards regression models among the failed 1^st^ operated eyes.

			Criterion			
	A		B		C	
	RR (95% Cl)	P value	RR (95% Cl)	P value	RR (95% Cl)	P value
Interval between TLE per month	0.98 (0.93–1.01)	0.20	0.98 (0.95–1.01)	0.23	0.98 (0.95–1.01)	0.14
Preoperative IOP per mmHg	1.02 (0.85–1.23)	0.83	0.94 (0.81–1.07)	0.36	0.96 (0.87–1.05)	0.39
Age per year	1.02 (0.94–1.12)	0.60	1.00 (0.93–1.06)	0.88	1.00 (0.95–1.06)	0.88
Type of glaucoma (XFG/POAG)	3.02 (0.23–92.8)	0.42	3.48 (0.47–34.5)	0.23	1.31 (0.31–5.93)	0.71
Lens status (pseudophakia/phakia)	0.12 (0.00–1.87)	0.13	0.31 (0.03–2.45)	0.27	0.50 (0.10–2.34)	0.38
Preoperative glaucoma medication per each	1.89 (0.39–12.1)	0.44	1.04 (0.42–2.59)	0.94	0.69 (0.29–1.51)	0.37

IOP = intraocular pressure; POAG = primary open-angle glaucoma; RR = relative risk; TLE = trabeculectomy; XFG = exfoliation glaucoma

## Discussion

The aim of the present study was to investigate whether the surgical outcomes of trabeculectomy were different between first operated eyes and second operated eyes. This retrospective study showed no significant differences in outcomes between the two eyes when all 84 patients were analyzed. However, among the patients who had successful trabeculectomies for their first operated eyes, the Kaplan–Meier survival curves showed significantly worse outcomes of the second trabeculectomy when the interval between trabeculectomies was 2 months or longer than when the interval was less than this. Furthermore, multivariable analyses confirmed that the longer interval between trabeculectomies was the significant prognostic factor of failure for the second trabeculectomy in patients with a first successful trabeculectomy. However, this association with the outcome of trabeculectomy for the second operated eye was not seen in the patients whose first trabeculectomy had failed. These data indicate that a longer interval between trabeculectomies can result in trabeculectomy failure for the second operated eye if the first trabeculectomy is successful.

There have been two large-scale retrospective studies about trabeculectomies for both eyes. Mietz et al. [15] analyzed 138 patients with both eyes trabeculectomized. Additional intervention occurred more frequently for the second operated eyes because of bleb encapsulation, although no significant difference was found between the eyes regarding postoperative IOP, the number of antiglaucoma medications taken, or the rate of surgical failure. In that study, 64.5% patients suffered from primary OAG or exfoliation glaucoma; the glaucoma types of the rest were developmental glaucoma, angle-closure glaucoma, or another secondary glaucoma. In some of their patients, surgical procedures, such as lens extraction, scleral buckling, retinal cryopexy, surgical peripheral iridectomy, or cyclocryocoagulation, had been performed in one or other eye before the trabeculectomy [17],[18]. These procedures cause conjunctival scarring, which could skew the surgical outcome of trabeculectomy for either eye [19],[20]. Jung et al. [16] analyzed 42 patients who underwent bilateral trabeculectomies by one expert surgeon. The glaucoma types of 88% of these patients were primary OAG or exfoliation glaucoma. Surgery with conjunctival manipulation was not performed in any eye before the trabeculectomy. In that study, early postoperative IOPs and bleb vascularity were significantly higher in the second operated eyes, whereas surgical success showed no significant difference between the eyes. The results of these two earlier studies appear to have a similar tendency, namely that the second trabeculectomy was associated with somewhat poorer outcomes, although Kaplan–Meier survival curves did not show significant differences between the eyes. As well as having some similarities to those previous reports, the present study is unique because it included patients at multiple centers with both eyes showing OAG, whose primary trabeculectomies were both performed by the same surgeon, and revealed significantly poorer outcomes for the trabeculectomies for the second operated eyes when these were performed >2 months after successful trabeculectomies in the first operated eyes.

At present, we cannot determine the reason why the longer interval after successful trabeculectomy resulted in a higher rate of trabeculectomy failure in the second eyes. Our hypothesis for the mechanism is that immunocompetent cells in the subconjunctival space recognize the intraocular antigens in the aqueous humor because of the scleral bypass created by the first trabeculectomy. The success of the first trabeculectomy offers, over the period of ≥2 months, long-term exposure of the intraocular antigen to the subconjunctival space. The enhanced immune reaction may cause bleb fibrosis when the second eye is treated with trabeculectomy. This hypothesis is consistent with there being no dependency of the failure of the second trabeculectomy on the length of time following a failed first trabeculectomy. The reduction in IOP because of trabeculectomy depends on the scleral bypass of the aqueous humor. A failed trabeculectomy is associated with failed filtration of aqueous humor via the scleral bypass, which could prevent immune communication between the intraocular antigens and the subconjunctival space in the first operated eye. These present data support the hypothesis that immune reaction to the intraocular antigen in the first operated eye may cause bleb failure in the second operated eye. Indeed, corneal transplantation graft failure frequently occurs in eyes in which glaucoma filtering surgery has been performed [12],[13],[14]. It is possible that immune communication between the intraocular antigens and the subconjunctival space could cause an adverse effect on the surgical outcome of additional surgery. A pain-related inflammatory chemokine was significantly increased in aqueous humor in the fellow eye after first-eye cataract surgery. This suggested there may be a sympathetic ophthalmic type uveitis in the contralateral eye after first-eye cataract surgery [21],[22].

Among the preoperative characteristics, BCVA was significantly worse in first operated eyes than in second operated eyes. Surgical priority seems to depend on the severity of visual acuity, visual field defect, IOP levels, and the number of medications taken. Previous reports have also shown the preoperative data indicating greater severity in first than in second operated eyes, such as having a more severe visual field defect [16] or worse visual acuity [15]. Despite the more severe preoperative data in the first operated eyes, these previous reports still showed poorer outcomes in the second operated eyes. Although we cannot completely exclude the possibility that a confounding factor in the preoperative data affected the outcomes, it is notable that second operated eyes with better visual acuity than first operated eyes showed poorer outcomes if the interval between the two trabeculectomies was long.

The present study has some limitations, mostly because of its multicenter, retrospective nature. First, we were unable to standardize surgical procedures (e.g., the concentration and duration of mitomycin C use, the size of scleral flap, and the number of scleral sutures) or postoperative procedures (e.g., laser suture lysis, ocular massage, and medications). To minimize intraoperative and postoperative bias, we recruited patients with both eyes operated by one surgeon. Second, we were unable to collect some clinical data. Conjunctival vascularity during the perioperative period and postoperative inflammation in the anterior chamber could affect bleb formation in the second operated eyes if an immune reaction after the first trabeculectomy was critical. Third, we could not compare bleb grading scores, including bleb vascularity, between the eyes. A previous study showed more intense vascularity in second operated blebs [16]. Fourth, in the present study, we could not evaluate the early postoperative IOP. A previous study showed that the difference in IOP between first and second operated eyes was greater in patients with a less than three week interval than in patients with longer intervals between trabeculectomies, so that they recommended the interval between trabeculectomies to be more than three weeks [16]. A further prospective study would be needed to clarify these limitations.

In conclusion, among patients with a successful first trabeculectomy, a longer interval before the trabeculectomy for the second eye is a significant prognostic factor for surgical failure of the second trabeculectomy, although this is not the case when the first trabeculectomy has failed. The results imply that if both eyes have a surgical indication, both should be treated with trabeculectomy within a short period of time. Further animal experiments are expected to reveal the involvement of immune communication because of bleb formation in the first operated eyes.
